# The Proposed United States Office of Management and Budget Rule for Managing Biomedical Research Grants and Cooperative Agreements Needs to Change

**DOI:** 10.4269/ajtmh.26-0409

**Published:** 2026-06-30

**Authors:** Philip J. Rosenthal, Jamie Bay Nishi, Albert I. Ko, David A. Fidock, Terrie E. Taylor

**Affiliations:** ^1^ Department of Medicine, University of California, San Francisco, California, USA;; ^2^ Chief Executive Officer, ASTMH, Arlington, Virginia, USA;; ^3^ Department of Epidemiology of Microbial Diseases, Yale School of Public Health, New Haven, Connecticut, USA;; ^4^ Instituto Gonçalo Moniz, Fundação Oswaldo Cruz, Salvador, BA, Brazil;; ^5^ Department of Microbiology and Immunology, and Center for Malaria Therapeutics and Antimicrobial Resistance, Division of Infectious Diseases, Department of Medicine, Columbia University Irving Medical Center, New York, NY, USA;; ^6^ College of Osteopathic Medicine, Michigan State University, East Lansing, Michigan, USA

The past 18 months have been extremely challenging for biomedical and public health research, due in large part to funding cuts and sweeping policy changes at US federal agencies. The latest challenge for biomedical researchers is new draft regulation from the US Office of Management and Budget (OMB), which was released on May 29, 2026. The proposed rule changes uniform guidance around federal financial assistance and will impact all grants and cooperative agreements funded by the US government. The new policy may sound well intended, ensuring good stewardship of taxpayer dollars, transparency, accountability, and oversight. However, several components of the 400+ page regulation would hinder rather than facilitate achievement of these objectives. Indeed, if the new regulation is adopted, there will be drastic changes to how the US government supports research, with tremendous and lasting damage to research programs. Several components of the regulation have already been operationalized via executive orders over the past 18 months. Those executive orders offered guidance; what is being proposed now is a set of binding rules. These rules will solidify the prior guidance, making it difficult to change or reverse it in the future. Specific concerns related to the OMB regulation are as follows.

First, a core feature of biomedical research support in the US, namely the primacy of peer review by expert scientists to guide funding decisions, will be demoted to a lower level “advisory” role. Currently, subject experts review and rank proposals using standardized, rigorous criteria. Under the new rule, funding decisions will be made by political appointees, based presumably not on scientific quality, but on “applicable law, the President’s policy priorities, and the national interest”. There is specific emphasis on metrics that measure the performance of “Gold Standard Science”, dictates to not fund projects that “promote anti-American values”, and assurance that awards “demonstrably advance the President’s policy priorities”. This is problematic, because the regulatory definition of what serves US national interests or advances “Gold Standard Science” is insufficiently clear, creating uncertainty for applicants and reviewers. US-supported biomedical science has led the world for generations, offering breathtaking advances that have improved the lives of Americans and others worldwide. It is deeply concerning to learn of a new regulation that will impede progress and weaken US leadership in this area.

Second, the proposed OMB regulation states that research grants can be terminated at any time, at the discretion of the government, if an award is determined to no longer align with agency priorities, program goals, or national interest. Two key features of US biomedical funding have been that grants include stable funding for relatively long periods, typically 4 to 5 years, and that funding is independent of the political priorities of a given US administration. Long-term funding allows groups to establish expert teams to pursue their research and enables trainees to conduct the long-term studies required for graduate and post-graduate training. Throwing uncertainty into this process and worse, disrupting projects midstream, will greatly hinder scientific progress. In clinical studies, abruptly halting a project after participants have consented raises ethical concerns.

Third, the OMB regulation prohibits research collaborations with scientists from a broad range of countries. The prohibition on foreign collaborations will cause major disruptions, especially for international infectious disease research, surveillance networks, outbreak investigations, and public health partnerships. The US government already has several restrictions in place related to collaborations with sanctioned countries; with the new regulation, a clear definition of unacceptable countries is not provided. A “domestic first” agenda will add difficulties even for collaborations with scientists working in countries closely allied with the US. The complexities of geopolitics are appreciated, but blocking the flow of ideas among all scientific experts and disrupting research in many countries harboring diseases of national and international relevance will only slow scientific progress.

Fourth, a chilling component of the OMB regulation states that a grant application may be denied due to an applicant’s membership in or affiliation with organizations that “undermine public safety or national security.” We fear that this wording, which echoes past assaults on the constitutionally protected First Amendment, from the Alien and Sedition Acts to the McCarthy era, could stymie research that is considered inconsistent with a given administration’s priorities, e.g. work on international equity, climate change, or public health for disadvantaged populations.

Fifth, proposed administrative changes, though seemingly technical and minor, are likely to greatly obstruct scientific progress. Demands for additional oversight have already led to major increases in the administrative burden of managing grants; ironically, these come from policymakers who extol the virtues of decreased administrative burdens. As an example, the US Department of Homeland Security E-Verify system must now be utilized for every employee and contractor working on a federal award, a particular burden for foreign research sites, where turnover of even non-scientist employees can grind research to a halt pending system checks.

Sixth, without express prior permission, federal funds will no longer be available to fund attendance at scientific conferences, subscriptions to scientific journals, or publication fees for scientific papers. Dissemination of scientific information is vital to all research. Indeed, without dissemination, research has little value. Researchers routinely rely on their grants to fund attendance at meetings where they communicate their findings to their peers, to provide access to scientific journals, and to publish their research. This is the very core of public accountability regarding research generated with public funds. The dissemination of peer-reviewed results and insights advances scientific knowledge in the public domain and is the imprimatur of gold standard science. Preventing this practice will severely limit scientists’ ability to share their findings and to translate these findings into the advances that have been the hallmark of American biomedical research for many decades.

As of this writing, the OMB regulation is only in draft form, with the opportunity for public comment for 45 days after its announcement. Considering the breadth of the proposed policy and the profound impacts of these new dictates, this period of public comment needs to be extended. It is vitally important that affected scientists voice their concerns. The American Society of Tropical Medicine and Hygiene (ASTMH) routinely represents the interests of its US and global membership and the tropical medicine/global health community at large, through interactions with the legislative and executive branches of the US government, and it will continue to do so in the context of the proposed rule changes.

We also urge members of the ASTMH, readers of its professional journal – the *American Journal of Tropical Medicine and Hygiene*, and all those interested in the future of US engagement in global health and the continued success of the US biomedical enterprise, to join us in speaking up.

A few ways to address concerns regarding the OMB regulation are as follows.

1. Submit a letter to OMB (Regulations.gov OMB-2026-0034 Docket) formally requesting an extension to the comment period. New regulations of such broad breadth and scope deserve careful review. An ASTMH submission on this topic can be found here.
2. Join an ASTMH member-only online dialogue on July 7, 2026, discussing the latest policy changes out of Washington, including the new OMB regulation.
3. Broadly engage and submit comments on how the proposed OMB changes would impact you and your work. Comments can be submitted anonymously. It is critical for OMB to receive unique submissions, so describe specific impacts to your work or field rather than copying text from others.
4. Reach out to your elected officials in Congress to share concerns about how the OMB rule, if enacted, will impact your work. It is particularly helpful to consider impacts in the context of the economy, jobs, health security, and the well-being of your state or district. Congress may have limited ability to act before the OMB rule is finalized, but legislators should be educated now on its sweeping impacts.
5. Beyond specific responses to the OMB regulation, highlight and explain your work and the importance of research on global health with your local community, including non-technical audiences.

The suggestions above are for the short term. It is critical to voice concerns about the new draft OMB regulation now. Moving beyond the short term, our times will remain challenging, and continued vigilance in the defense of science is critical.

